# Scientists’ Assessments of Research on Lactic Acid Bacterial Bacteriocins 1990–2010

**DOI:** 10.3389/fmicb.2022.908336

**Published:** 2022-06-03

**Authors:** Laura D. Martinenghi, Jørgen J. Leisner

**Affiliations:** Department of Veterinary and Animal Sciences, Faculty of Health and Medical Sciences, University of Copenhagen, Frederiksberg, Denmark

**Keywords:** antibiotic, antimicrobial, biopreservation, lantibiotic, nisin, pediocin, bacteriocin, lactic acid bacteria

## Abstract

The antimicrobial activity of bacteriocins from lactic acid bacteria has constituted a very active research field within the last 35 years. Here, we report the results of a questionnaire survey with assessments of progress within this field during the two decades of the 1990s and the 2000s by 48 scientists active at that time. The scientists had research positions at the time ranging from the levels of Master’s and Ph.D. students to principal investigators in 19 Asian, European, Oceanian and North American countries. This time period was evaluated by the respondents to have resulted in valuable progress regarding the basic science of bacteriocins, whereas this was not achieved to the same degree with regard to their applications. For the most important area of application, food biopreservation, there were some success stories, but overall the objectives had not been entirely met due to a number of issues, such as limited target spectrum, target resistance, poor yield as well as economic and regulatory challenges. Other applications of bacteriocins such as enhancers of the effects of probiotics or serving as antimicrobials in human clinical or veterinary microbiology, were not evaluated as having been implemented successfully to any large extent at the time. However, developments in genomic and chemical methodologies illustrate, together with an interest in combining bacteriocins with other antimicrobials, the current progress of the field regarding potential applications in human clinical microbiology and food biopreservation. In conclusion, this study illuminates parameters of importance not only for R&D of bacteriocins, but also for the broader field of antimicrobial research.

## Introduction

The antimicrobial activity of lactic acid bacterial (LAB) bacteriocins—especially class I (containing lanthionine and β-methyllanthionine) and class II (small, heat-stable) peptides (1990s classifications applied)—has constituted a very active research field within the last 35 years. Their application as food biopreservatives has been an important objective; as a partially purified compound (nisin), a component in fermentates (pediocin PA-1) or by *in situ* production by an added culture (Holzapfel et al., 1995; Stiles, 1996; Ennahar et al., 2000; Cleveland et al., 2001; Twomey et al., 2002; Cotter et al., 2005; Drider et al., 2006; O’Connor et al., 2020). Bacteriocins have also been examined for applications in veterinary microbiology and human clinical microbiology (Twomey et al., 2002; Servin, 2004; Drider et al., 2006; Hassan et al., 2012; Cotter et al., 2013; Yang et al., 2014; Alvarez-Sieiro et al., 2016; Chikindas et al., 2018; O’Connor et al., 2020; Soltani et al., 2021).

Investigations of bacteriocin structures and biosynthesis, mode of action, secretion, and genetic regulation, e.g., by quorum sensing, have constituted important parts of this field (Nes et al., 1996; Kleerebezem et al., 1997; Kuipers et al., 1998; Sahl and Bierbaum, 1998; Ennahar et al., 2000; Cintas et al., 2001; Kleerebezem and Quadri, 2001; Eijsink et al., 2002; Garneau et al., 2002; Héchard and Sahl, 2002; Fimland et al., 2005; Nissen-Meyer et al., 2009). On the other hand, the ecological functions of LAB bacteriocins have only attracted relatively limited attention, although their role appears to differ from colicins that constitute an established model for our understanding of the ecology of bacterial antimicrobial peptides (Dykes and Hastings, 1997; Eijsink et al., 2002; Leisner and Haaber, 2012; Snyder and Worobo, 2013).

Historically, research on LAB bacteriocins began with empirical screenings of antimicrobial activity by producer cultures, typically originating from foods, starting in earnest in the late 1980s and the early 1990s. This research typically focused on GRAS species among the bacteriocinogenic LAB but also on other potential bacteriocin producers within the Firmicutes and other phyla (Héchard and Sahl, 2002; Arnison et al., 2013; Acedo et al., 2018). During the 1990s and 2000s, it became apparent that resistance by target organisms was a common phenomenon (Montville et al., 1995; Gravesen et al., 2002; Cotter et al., 2005, 2013; Drider et al., 2006; Naghmouchi et al., 2007). This situation, combined with the fact that the antimicrobial spectra of many bacteriocins are relatively narrow, promoted research into synergy effects by combining them, also with other antimicrobial compounds or even phages (Lüders et al., 2003; Mathur et al., 2017; Rendueles et al., 2022). Research has also been devoted to synthetic or bioengineered bacteriocins (Ennahar et al., 2000; Ongey and Neubauer, 2016; Soltani et al., 2021).

Here, we present the accounts of scientists involved in research on bacteriocins from 1990 to 2010 in the form of their responses to a questionnaire probing their choices of producer and target organisms, types of bacteriocins, studies of the mode of action and underlying genetics, intended applications, and their opinion then and now on whether research objectives were met, both in relation to their own studies and to the field as such. This study serves as an informal supplement to a large number of reviews and opinions that have been published on LAB bacteriocins over the last 30 years.

## Materials and Methods

### Bibliometric Analyses

A topic search was done using Web of Science (WoS) with the key terms “Lactic acid bacteria AND bacteriocin(s)” for 1990–2010. The search returned 1,504 hits with information on numbers of articles, reviews, letters, and proceedings papers and information on WoS categories, authors, and institutions. The search also resulted in a full record for all 1,504 publications as well as citations for the individual publications. Citation windows (10 years) were manually extracted and number of patents were extracted from Scopus (Elsevier).

### Questionnaire

Respondents were contacted by e-mails containing individual links to the online questionnaire hosted by Userneeds (Copenhagen, Denmark), which supplies web-based questionnaire surveys. Individual answers were kept anonymously according to existing GDPR rules.

Potential participants were selected by a combination of personal knowledge of the field at the time by one of us (JJL) and by identifying individual research groups from the bibliometric search using the terms “lactic acid bacteria” AND “bacteriocin(s)” for publications from 1990 to 2010. Respondents were then found among the 20 institutions with the most publications, including researchers with high or low amounts of publications on the topic. In addition, a number of respondents was selected from additional institutions. In general, the selection contained a range of researchers with variations in research outputs for bacteriocins. Among the 30 researchers with the highest output from 1990 to 2010, 22 were contacted and 12 completed the questionnaire. Overall, 94 researchers were contacted, with 54 responding and 48 completing the questionnaire.

The questionnaire (Supplementary Datasheet 1) was organized into four sections: profiles (eight questions), details on respondents’, research on bacteriocins (22 questions), respondents’ memories of their opinions during the 1990s and 2000s on the research (11 questions), and current opinions of respondents on whether research objectives were reached at the time (seven questions), in total 48 questions (question 9 was divided into two; see Supplementary Datasheet 1 for questionnaire text). In the first two sections, most questions were open, but in a few cases, we used a five-point scale for answers. In the last two sections, we employed a seven-point scale for answers including the following terms: completely agree, strongly agree, agree, neither agree/disagree, somewhat disagree, strongly disagree, and completely disagree. Re-analysis of results pooling the responses into three categories—agree, neither agree nor disagree, and disagree—gave similar results. It was possible to add comments to nearly all questions. A total of 29 out of 48 respondents used this possibility, with 10 respondents (all associated with different institutions) adding at least four comments each. Comments are listed in Supplementary Datasheet 2.

## Results

### Profile of Respondents and Details on Their Research on Bacteriocins

The respondents represented a broad spectrum of researchers in the field from 1990 to 2010, as reflected by their information on age, years of research in the field, and job positions, ranging from Master students to PI’s [Question (Q) 1–8]; Supplementary Table 1. The number of active researchers among the respondents was higher in the 1990s (40) than in the 2000s (34). The numbers of PhD. students and post-docs were highest in the 1990s (17 and 10 compared to 3 and 6, respectively in the 2000s), whereas the numbers of PI’s, Professors, and Associate Professors were higher in the 2000s (results not shown). The data gave the impression of a cohort and input from respondents at Ph.D. and post-doc levels in the latter decade were therefore underrepresented.

Among the respondents, 19 (26.8%) were among 71 researchers with at least 10 publications in the 1990s and 2000s found using the search term “Lactic acid bacteria AND bacteriocin(s).” Two respondents were among the top five regarding publication output. An additional 15 respondents had between five and nine publications, whereas the remaining 14 had below five publications. Individual searches revealed higher numbers of publications on bacteriocins for some respondents as the search term applied would not cover all research aspects.

Respondents were associated with laboratories in 19 countries, including Brazil, Canada, Japan, Malaysia, New Zealand, South Africa, the United States, and 12 European countries (Q8; Supplementary Table 2). Countries with the most respondents included Canada, France, Ireland, Netherlands, Norway, and the United States.

The researchers had worked with a wide range of LAB bacteriocin producers, including especially the genera *Carnobacterium*, *Enterococcus*, and *Lactobacillus* (including some of the new genera created after a recent taxonomic revision; Zheng et al., 2020), *Lactococcus* and *Pediococcus* (Q16; Supplementary Table 3). In addition, a number of bacteriocin producers other than LAB had also been examined by some researchers, especially species belonging to *Bacillus* or *Staphylococcus* (Q17; Supplementary Table 4). The number of respondents who had worked on probiotics amounted to 18 out of 48 (37.5%; Q21).

A number of different non-pathogenic LAB target strains were included in research on antimicrobial spectra (Q9a; Supplementary Table 5). Among the Gram-positive foodborne pathogens, especially *Listeria monocytogenes* followed by *Staphylococcus aureus*, *Enterococcus* spp., *Bacillus* spp., and *Clostridium* spp. were used as targets (Q9b; Supplementary Table 6). Gram-negative foodborne pathogens were also included by many respondents as target strains, but it is safe to assume, that they under most conditions, were not sensitive toward the majority of bacteriocins (Q10; Supplementary Table 6). A selection of Gram-positive foodborne spoilage organisms were examined by some respondents (Q11; Supplementary Table 6). The majority of applied research on bacteriocins was devoted to biopreservation of foods, especially (in the following order) meat, milk/dairy, and seafood (Q18; Supplementary Table 7).

Regarding Gram-positive and Gram-negative human clinical species as target strains, some were also listed under foodborne pathogens by a number of respondents [e.g., *Escherichia coli*, *Salmonella, S. aureus*, and *Streptococcus pyogenes* (but see Falkenhorst et al., 2008); Q12, 13]. A minority of respondents mentioned some additional pathogens: *Cutibacterium acnes*, *Clostridium* (now Clostridiodes) *difficile*, *Streptococcus agalactiae*, *Streptococcus mutans*, *Streptococcus pneumoniae*, *Streptococcus pyogenes* (all Gram-positive; 10 respondents), *Acinetobacter baumannii*, *Helicobacter pylori*, *Klebsiella pneumoniae*, *Legionella pneumophila*, *Pseudomonas aeruginosa* (five respondents), and antibiotic-resistant variants of pathogenic bacteria (MRSA: Methicillin Resistant *Staphylococcus aureus*; ESKAPE: *Enterococcus faecium*, *Staphylococcus aureus*, *Klebsiella pneumoniae*, *Acinetobacter baumannii*, *Pseudomonas aeruginosa*, and *Enterobacter* spp.; VRE: Vancomycin Resistant Enterococci; four respondents; Q12, 13). A minority of respondents also worked with target strains from species important in veterinary microbiology, including *Enterococcus faecium*, *Enterococcus faecalis*, *Lactococcus garvieae* (fish pathogen), *Listeria ivanovii*, *Listeria monocytogenes*, *Staphylococcus aureus* (mastitis research), *Streptococcus* spp. (mastitis research) including *Streptococcus uberis*, *Streptococcus dysgalactiae* (all Gram-positive; 14 respondents), and *Aeromonas* spp. including *Aeromonas salmonicida* (fish pathogen), *Tenacibaculum* spp. (fish pathogen), *Vibrio* spp., and *Yersinia* spp. (all Gram-negative; 11 respondents) in addition to *Mycobacterium avium* subsp. *paratuberculosis* (one respondent; Q14, 15).

**Figure 1:**
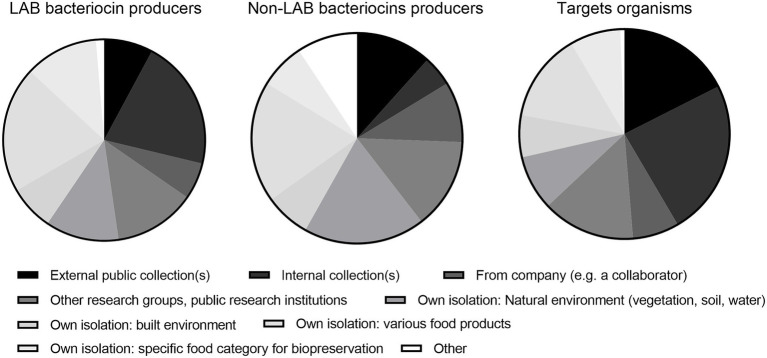
Sources of strains of lactic acid bacterial (LAB) bacteriocin producers (47 respondents), non-LAB producers (26 respondents), and target strains (48 respondents; Q22–24).

The respondents obtained bacteriocin producers and target strains from many sources, but external (public) and internal collections constituted a higher proportion of sources for target strains (Q22–24; Figure 1). It might be speculated that the variety among target strains from culture collections was relatively low but further research is needed to clarify this issue.

**Figure 2:**
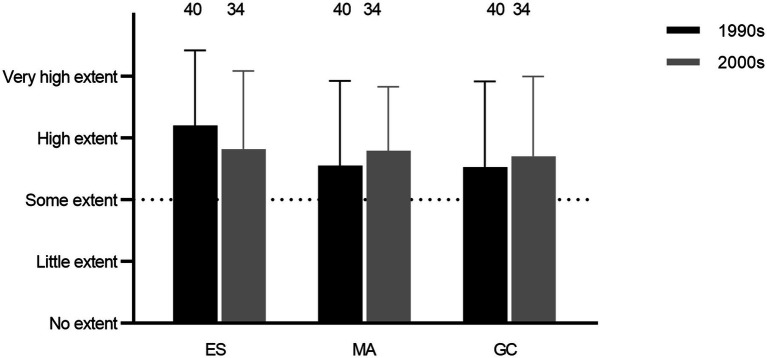
Average degrees of research activities in the 1990s and 2000s involving empirical screenings (ES), studies of mode of action (MA), and genetic characterization (GC; Q25, 26, and 28).

**Figure 3:**
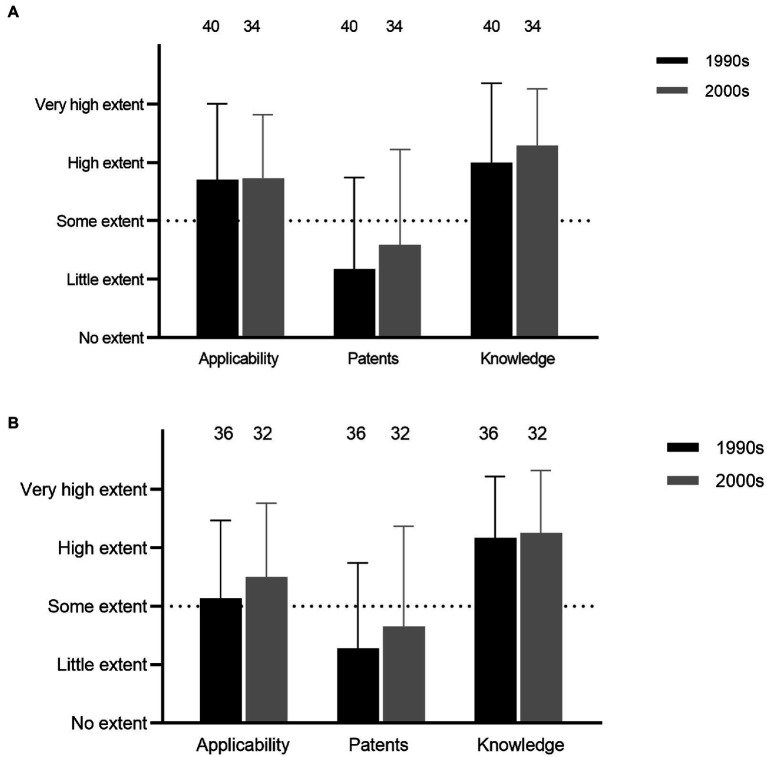
Objectives for research on mode of action **(A)** and genetic characterization (**B**; Q27 and 29).

Empirical screenings, mode of action, and genetic characterization of expression all constituted relatively high degrees of research activities among the respondents (Q25, 26, and 28; Figure 2). Further, for both studies of mode of action and genetic characterization, obtaining basic knowledge was the most important objective, followed by examining applicability, whereas the issue of obtaining patents was not perceived as important (Q27 and 29; Figure 3).

**Figure 4:**
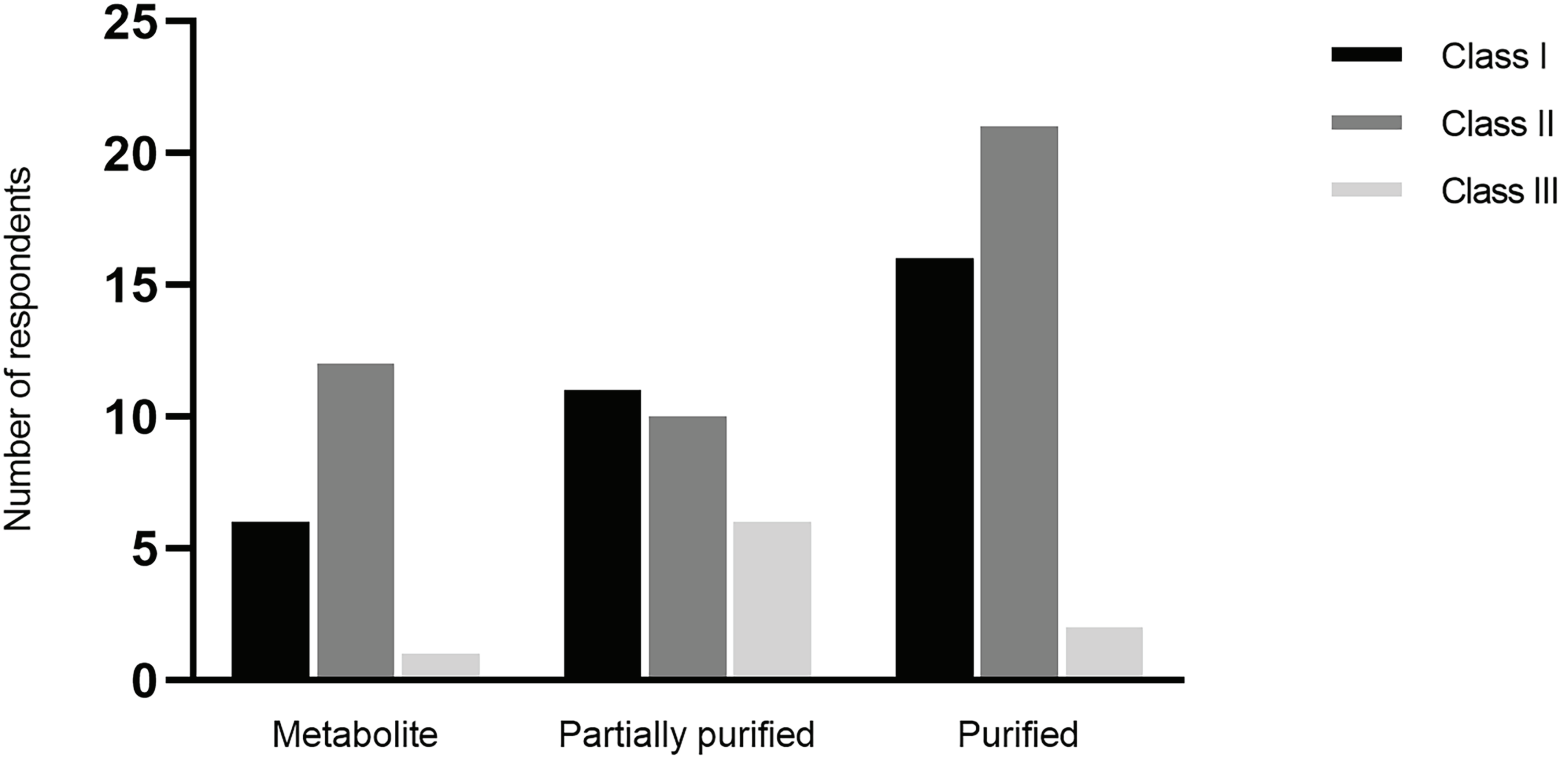
Class and level of purification of bacteriocins included in respondents’ research (Q19).

The research focused on class I and class II bacteriocins, mostly as purified or partially purified compounds, whereas class III bacteriocins (all classes as defined in the 1990s) attracted less attention (Q19 and 20; Figure 4). Overall, the respondents had worked with a broad range of both LAB and non-LAB bacteriocins (comments 1–17 and 18–35, respectively).

### Details on Opinion on the Field of Research and Research Objectives

The respondents were asked about their opinion on the number of research groups with LAB bacteriocins as one of the primary topics in the 1990s and the 2000s. These were difficult questions to answer and only a few respondents suggested numbers that varied widely; average values were around 60 and 55 research groups for the 1990s and 2000s, respectively (Q30, 31; comments 65–73). The bibliometric data showed that relatively few institutions and researchers had significant higher publication outputs for “Lactic acid bacteria AND bacteriocin(s)” with Norwegian University of Life Sciences (four of the top 20 researchers with highest publication outputs within the topic), Stellenbosch University (two researchers), National Research Institute for Agriculture, Food and Environment (INRAE), Vrije Universiteit Brussel (two researchers), and University of Alberta (two researchers) in the top five.

**Figure 5:**
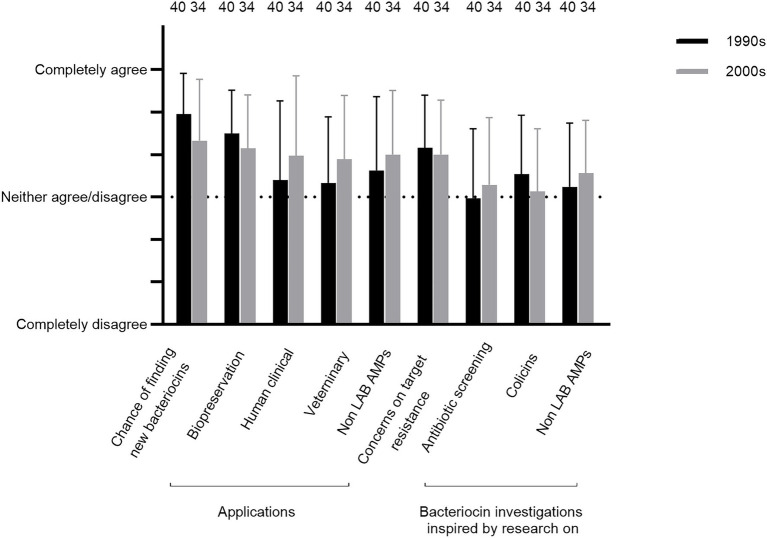
Respondents’ memories of their opinions in the 1990s and/or the 2000s about whether the chances were high regarding finding new bacteriocins including compounds useful for various applications and whether they were inspired by related research fields (Q32–40).

Questions Q32–40 were concerning respondents’ memories of opinions about the different aspects of the bacteriocin research field back in the 1990s and/or the 2000s. On average, the researchers agreed or strongly agreed that the chances of finding new bacteriocins were high, especially in the 1990s (Q32; Figure 5; but see the variations in answers provided by comments 46–48, 50–51, 152, 154, and 159). The chances of finding new bacteriocins with potential practical applications for biopreservation of foods were perceived positively (Q33; Figure 5), although some comments did not support this (86–87). Further, the researchers did not agree to the same extent that the chances were high of finding bacteriocins for practical applications for the treatment of infections by human clinical pathogens or veterinary pathogens in the 1990s (Q34 and 35; Figure 5; comments 111–115 and 122–124). A similar pattern was observed for the evaluation of whether the chances were high for finding new practical applicable antimicrobial peptides from other organisms than lactic acid bacteria, including eukaryotic organisms (animals, plants, and/or fungi) for treatment of infections by human clinical pathogens (Q36; Figure 5). It should be noted that the degree of variation in responses was higher for Q34–36 than for Q32 and Q33. One factor that weighs in regarding the evaluation of chances for application of bacteriocins was the issue of target resistance, which the researchers to some extent agreed was a cause for concern (Q37; Figure 5; comments 62–64).

Regarding inspiration, the researchers neither agreed nor disagreed that previous research on antibiotics and colicins provided such a source (Q38, 39, Figure 5, comment 74–76). The same outcome was noted regarding inspiration from contemporary screening studies in the related field of new animal, plant, and/or fungal antimicrobial peptides (Q40, Figure 5).

**Figure 6:**
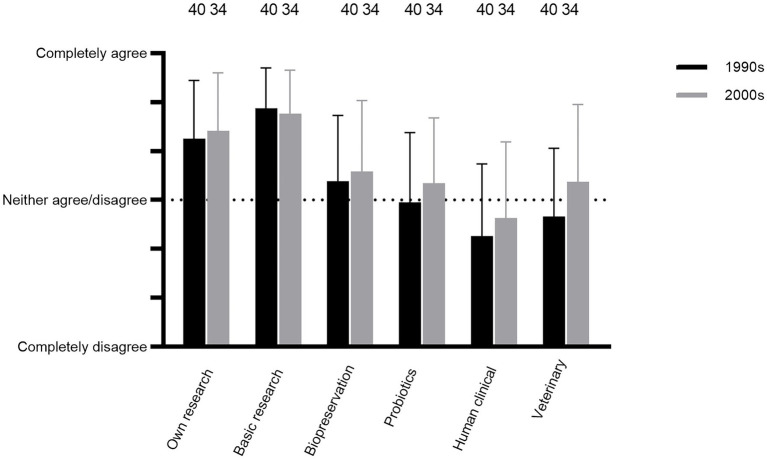
Respondents’ current opinions about whether LAB bacteriocin research in the 1990s and 2000s fulfilled the objectives (Q41–46).

Questions Q41–46 were concerning current opinions by respondents on whether bacteriocin research reached the objectives in the 1990s and/or the 2000s. The researchers, on average, agreed that bacteriocin research met the personal objectives set by the individual researcher from 1990 to 2010, including that the field presented an important training ground for researchers early on in their careers (Q41; Figure 6; comments 77–81). This positive outcome was even more pronounced regarding opinions on whether the overall field of research on lactic acid bacterial bacteriocins met the objectives in terms of contributing to basic knowledge on bacteria antagonism (Q42; Figure 6; comment 82 but see comments 83–84).

However, there was, on average, some skepticism about whether the bacteriocin research had met the objectives from 1990 to 2010 in the applied aspects (Q43–46; Figure 6). The best outcome was perceived for food biopreservation (Figure 6, comments 88–97). Views on probiotic applications were relatively similar to what were perceived for biopreservation, whereas applications for veterinary microbiology and human clinical microbiology were viewed somewhat less favorable (Figure 6; see comments 98–110 for probiotics; 116–119 for human clinical microbiology and 125 for veterinary microbiology).

An additional question (Q47) inquired whether the bacteriocin research field has benefitted from developments in sequence-based methodology and associated bioinformatics. These developments were generally perceived very positively (comments 126–162), although it was noted that additional research was required to illuminate functional and applied aspects (comments 129, 137, 141–142, 149, 151, 152, 154, 156, and 159).

At the end of the questionnaire, the respondents were encouraged to add any additional comment/memory/insight in relation to their bacteriocin research. Those comments were allocated to the specific questions they addressed.

## Discussion

**Figure 7:**
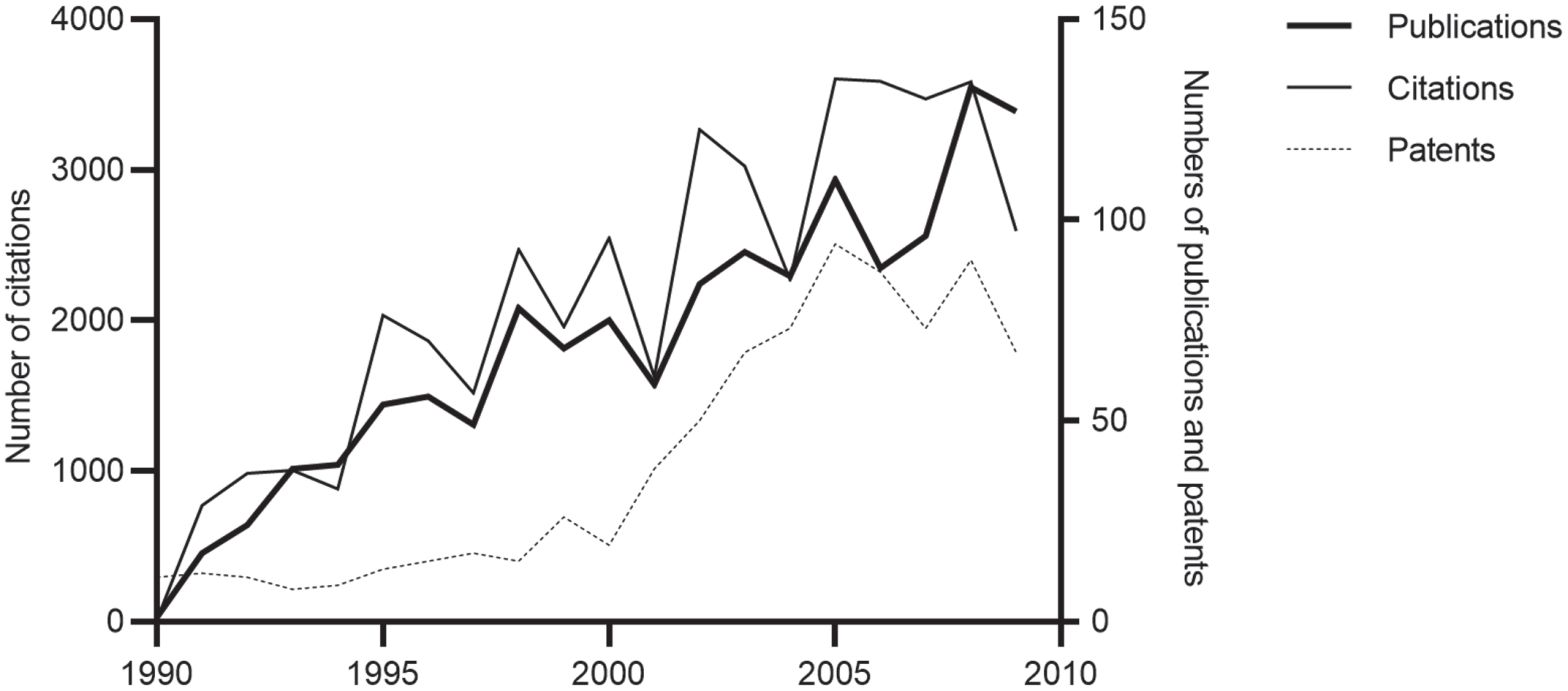
Bibliometric data on number of publications, citations, and patents 1990–2009.

Examples of lactic acid bacterial bacteriocins have been known since the 1960s (nisin since the late 1920s/early 1930s), but they first became an active area of research from the late 1980s and onwards (Figure 7; De Klerk and Coetzee, 1961; Brock et al., 1963; Delves-Broughton et al., 1996), the period of time covered by this questionnaire study. Some respondents indicated an increase in research groups in the 1990s but stagnation or even a decrease in the 2000s (Comments 65–73). The total research output increased during both decades, but the number of citations leveled during the latter part of the 2000s (Figure 7). Interestingly, the number of patents shows another trend with relatively few granted in the 1990s followed by a sharp increase in the first half of the 2000s, although this issue was not an important motivator for research (Figures 3, 7). The increase in patents over time agrees with the findings by López-Cuellar et al. (2016), although their overall number of patents were substantially lower.

Two classes of bacteriocins, class I—lantibiotics, containing lanthionine and β-methyllanthionine residues and sometimes other unusual residues due to post-translational modifications and class II—small, heat-stable peptides, were the focuses for research. In contrast, larger antimicrobial class III proteins received less attention. It should be noted that the classification of bacteriocins has been a focus for revision with subsequent more elaborate classification systems and transfer between classes as a result. Thus, cyclic peptides were characterized as class II bacteriocins before 2010 but are now considered as posttranslational modified bacteriocins (Franz et al., 2007; Zouhir et al., 2010; Martin-Visscher et al., 2011; Rea et al., 2011; Cotter et al., 2013; Snyder and Worobo, 2013; Gabrielsen et al., 2014; Alvarez-Sieiro et al., 2016; Acedo et al., 2018; Chikindas et al., 2018).

The respondents were generally not inspired by earlier screening efforts for colicins or natural product antibiotics during the 1940s and 1950s (Figure 5, comments 74–76). However, that earlier epoch gave some potential important lessons regarding obstacles in antibiotic screening programs worth re-considering in the context of bacteriocins, including the risk of rediscovery, resistance among target organisms and potential toxicity (Peláez, 2006; Silver, 2011; Lewis, 2013; Genilloud, 2014; Wright, 2014; Katz and Baltz, 2016; Aminov, 2017; Abouelhassan et al., 2019; Leisner, 2020). Further, there is some similarity with some antibiotics in routes of activities (comment 54).

The underlying motivation for detailed investigation of antibacterial systems may frequently be found in a desire to explore fundamental aspects as much or even more than examining their potential application. The mode of action of colicins constitutes an early example of this issue as they provided an insight into the functionality of membranes and membrane proteins as described by Luria (1984), one of the pioneers in that field. Overall, the responses in this study agree with the importance of basic science as a major motivator for bacteriocin research (Figures 5, 6).

Fundamental aspects of the research involved initial screenings for bacteriocinogenic cultures and subsequent investigations of promising bacteriocinogenic systems, including their antimicrobial spectra, structure and biosynthesis, mode of action, regulation of expression and secretion (Nes et al., 1996; Ennahar et al., 2000; Cintas et al., 2001; Eijsink et al., 2002; Garneau et al., 2002; Héchard and Sahl, 2002; Fimland et al., 2005; Nissen-Meyer et al., 2009; Snyder and Worobo, 2013; Ongey and Neubauer, 2016). In addition, genetic characterization of expressions of bacteriocins also gave insights in relation to the field of bacterial quorum sensing (Kleerebezem et al., 1997; Kuipers et al., 1998; Kleerebezem and Quadri, 2001). Overall, these fundamental studies were evaluated as having obtained a relatively high degree of success in the 1990s and 2000s (Figures 5, 6). However, some respondents expressed doubts regarding how much natural variation exists among bacteriocins and whether that had a negative impact on the discovery of novel compounds already in the 2000s (comments 48, 50, 51, 152, 154, and 159).

One fundamental area that, to some extent, was overlooked concerns the ecology of class I and II bacteriocins, as they do not readily fit into the ecological functionality described for colicins (Dykes and Hastings, 1997; Eijsink et al., 2002; Kirkup and Riley, 2004; Leisner and Haaber, 2012; Snyder and Worobo, 2013; comment 83). Their utilization as signals have been proposed, a function that mirrors previous suggestions for antibiotics (Linares et al., 2006; Snyder and Worobo, 2013; Chikindas et al., 2018). Among other possibilities can be mentioned that bacteriocins may mediate a supply of nutrients from lysed target cells (Leisner and Haaber, 2012) or facilitate the transfer of DNA *via* transformation (Snyder and Worobo, 2013). Further research would be relevant for the broader area of microbial interactions and may support application of bacteriocins in food systems (Gálvez et al., 2007; Ramia et al., 2020).

Class I and II bacteriocins were perceived early on to have potential for application as “natural” food preservatives, either by addition of bacteriocinogenic cultures or bacteriocins, e.g., in the form of encapsulation (Holzapfel et al., 1995; Stiles, 1996; Cleveland et al., 2001; Cotter et al., 2005; Drider et al., 2006; Boualem et al., 2013; Chikindas et al., 2018; O’Connor et al., 2020). In addition, bacteriocinogenic cultures were potentially useful in food technological applications such as for acceleration of cheese ripening (Oumer et al., 2001). Cooked or fermented meat products, dairy products, and (lightly preserved) fish products constituted the main food categories among the respondents for potential application of biopreservation (Supplementary Table 7). Foods served as the obvious choice for isolating potential bacteriocinogenic cultures for this purpose (Hastings and Stiles, 1991; Lewus et al., 1991; Garver and Muriana, 1993; Ruiz-Barba et al., 1994; Jones et al., 2008). Biopreservation was evaluated by respondents as having obtained a moderate degree of success in the 1990s and 2000s, although narrow target spectra, resistance, sensitivity to proteases, low level of production and legislation all presented challenges, as detailed below (Figures 6, 7; comments 86–94, 96–97, and 153).

In addition, bacteriocins were of interest as enhancers of probiotic actions, including fish probiotics in aquaculture (Ringø and Gatesoupe, 1998; Desriac et al., 2010; O’Connor et al., 2020). Further, they were considered as potential antimicrobial compounds in relation to veterinary and human clinical bacteriology—especially targeting antibiotic-resistant pathogens—but also in virology in addition to serving as potential anti-cancer agents (Wachsman et al., 2003; Servin, 2004; Drider et al., 2006; Montalbán-López et al., 2011; Hassan et al., 2012; Yang et al., 2014; Alvarez-Sieiro et al., 2016; Chikindas et al., 2018; Soltani et al., 2021). The bacteriocinogenic cultures were sometimes isolated from the same source for which they were intended for (veterinary) application (comment 38). The degree of success of these applications in the 1990s and 2000s was a subject of critical evaluation by some of the respondents in this study due to, e.g., the issue of toxicity in relation to systemic applications (Figures 5, 6; comments 16, 98–119, and 122–125). Biomedical applications against biofilms present another possible application (Bower et al., 2001; Mathur et al., 2017). This topic was not mentioned in the questionnaire and it was not commented upon by respondents. Further, bacteriocins found relatively few applications in a veterinary context except for topical applications such as mastitis (Ross et al., 1999; comments 122–125). Finally, a few comments mention the application of bacteriocins as feed additives, which are of interest in the light of controversies concerning the use of antibiotics for this purpose (Kirchhelle, 2018; Silveira et al., 2021; comments 120–121).

The relatively narrow antimicrobial spectra for many bacteriocins might be desirable in a human therapeutic context to minimize damage to the commensal human microbiota (Cotter et al., 2013). However, this would not be the case for many biopreservative applications. Combinations of bacteriocins with food-grade antimicrobials/stressors is a potential solution in that context (Gálvez et al., 2007; Mathur et al., 2017). Bacteriocins typically only inhibit related taxons (Eijsink et al., 1998, comment 84) and then frequently only some strains within a given species (Rasch and Knøchel, 1998; Carl et al., 2004), an issue that requires careful selection of a wider panoply of target strains in screening scenarios. The respondents informed on a higher usage of external and internal culture collections as a source for target strains compared with producer strains. With a bit of caution, this might be interpreted as perhaps involving a relatively narrow common range of target strains, making it more difficult to accurately estimate the frequency of resistance within a given target species (Figure 1).

In addition, as revealed by a few comments by the questionnaire respondents, resistance development among target organisms represented a challenge that was addressed by some research groups in the 1990s and onwards (Gravesen et al., 2002; Cotter et al., 2005; Naghmouchi et al., 2007, comments 62–64). Resistance had, however, a potential positive element in an explorative context, somewhat similar to dereplication screening efforts in searches for antibiotics (Leisner, 2020). Thus, target resistance to known bacteriocins was employed as a selection method to find new bacteriocins (comment 61).

Overall, a limited number of bacteriocins were introduced for applications from 1990 to 2010. Partially purified nisin and pediocin PA-1 (in the form of an added fermentate) were two commonly applied bacteriocins for food biopreservation at the time. They were, however, both discovered, and in the case of nisin, applied before the 1990s (Frazer et al., 1962; Bhunia et al., 1988; Delves-Broughton et al., 1996; Stiles, 1996; Rodríguez et al., 2002; Cotter et al., 2005; O’Connor et al., 2020). Thus, some of the few low-hanging fruits were found early on, and it was difficult to find new compounds with enhanced desired properties relative to the initial compounds (comments 48, 51, 152, 154, and 159). In this context, the phenomenon of rediscoveries of well-known bacteriocins represented an additional challenge (comment 46). This situation was not too different from the outcome of screening of natural product antibiotics in the 1940s and 1950s (Greenwood, 2008; Silver, 2011; Lewis, 2013; Wright, 2014; Leisner, 2020).

One additional important obstacle to the application of bacteriocins, both in relation to food biopreservation as well as in relation to medical and veterinary applications, is presented in the form of legislative demands such as bacteriocins should be safe to consume, and bacteriocinogenic cultures should also not contain genes encoding virulence factors or antibiotic resistance (Montville et al., 1995; Stiles, 1996; O’Connor et al., 2020). This made the use of enterococci for biopreservation a topic for discussion already in the 1990s (Jett et al., 1994; Franz et al., 1999; for a more positive account, see also Moreno et al., 2006). Finally, although the antimicrobial activity of bacteriocinogenic cultures might be improved by, e.g., combining the genetic systems for different bacteriocins in one organism or construction of strains that can produce the compounds in a heterologous manner (Hanlin et al., 1993; Ennahar et al., 2000; O’Sullivan et al., 2003), genetically modified LAB is still a controversial issue that meets regulatory demands (Plavec and Berlec, 2020).

Another obstacle at the time was represented by the difficulties in obtaining funding for searching for new bacteriocins (comments 85, 93–94, and 138). This might partially have been due to the lack of enthusiasm by some of the players in the food industry (comment 96, 97).

Overall, the objectives for research into bacteriocin applications in the 1990s and 2000s were only met to a partial degree, even within the area of food biopreservation, which constituted the single most important objective. The research did, however, provide important fundamental insights useful for the next ongoing phase that in addition to food biopreservation or probiotics focuses on potential medical applications of a wider range of antimicrobial peptides. These include, among others, post-translationally modified LAB and non-LAB peptides (RiPPs, among them class I bacteriocins), e.g., in combination with other antimicrobials (Arnison et al., 2013, Cotter et al., 2013; Yang et al., 2014; Alvarez-Sieiro et al., 2016; Mathur et al., 2017; Perez et al., 2018; O’Connor et al., 2020; Chen et al., 2021; Soltani et al., 2021). The introduction of genomics, including bioinformatics software already during the 2000s, such as BAGEL and antiSMASH (comments 146 and 149) has been important (de Jong et al., 2006, 2010; Medema et al., 2011; Nes, 2011; van Heel et al., 2013; several of the comments 126–162). This approach serves as a mean for dereplication in screenings (comment 149). There were, however, some notes of caution among respondents, as functional and applied aspects are not always easy to decipher from obtained sequences (Eijsink et al., 2002; comments 129, 137, 141–142, 151, 152, 154, 156, and 159) and could be supplemented with other approaches such as mass-spectrometry to characterize modification patterns of RiPPs (comment 149).

An important aspect not covered in the questionnaire concerns the chemical approach by targeted engineering of new peptides. This is an area that has attracted research from the 1990s and up to now but has been met with some challenges (Fimland et al., 1996, Ongey and Neubauer, 2016; comments 138, 149, 150, and 152). However, this scenario, if implemented, to some degree resembles the shift in antibiotics R&D during the 1960s from large screenings of natural compounds to the construction of semisynthetic variants (Leisner, 2020).

In conclusion, this questionnaire survey illuminates the formative years of research on class I, II, and III bacteriocins from lactic acid bacteria and other sources. The research was evaluated by respondents to have resulted in valuable progress regarding the basic science of bacteriocins, and there were some success stories within the area of food biopreservation (see e.g., Chikindas et al., 2018). However, issues such as limited target spectrum, target resistance, sensitivity to proteases, poor yield, and economic and regulatory challenges limited the overall success of this approach. Applications also met difficulties in relation to human clinical and veterinary microbiology. The findings in this study highlight how progress in the studies of bacteriocins depended on a number of parameters, some of which are also of interest in the broader field of antimicrobial research.

## Data Availability Statement

The raw data supporting the conclusions of this article will be made available by the authors, without undue reservation. Any requests should be directed to the corresponding author.

## Ethics Statement

Ethical review and approval were not required for the study on human participants in accordance with the local legislation and institutional requirements. Written informed consent for participation was not required for this study in accordance with the national legislation and the institutional requirements.

## Author Contributions

JL designed the study. LM and JL performed the research and wrote the manuscript. All authors contributed to the article and approved the submitted version.

## Funding

This study was funded by a research grant from the Norwegian Research Council (grant 314490-FORSKER20).

## Conflict of Interest

JL has a minor share in a company that sell probiotic cultures.

The remaining author declares that the research was conducted in the absence of any commercial or financial relationships that could be construed as a potential conflict of interest.

## Publisher’s Note

All claims expressed in this article are solely those of the authors and do not necessarily represent those of their affiliated organizations, or those of the publisher, the editors and the reviewers. Any product that may be evaluated in this article, or claim that may be made by its manufacturer, is not guaranteed or endorsed by the publisher.
