# Supplementary material for: Scientists’ Assessments of Research on Lactic Acid Bacterial Bacteriocins 1990–2010
Source: Front Microbiol. 2022 Jun 3;13:908336. doi: 10.3389/fmicb.2022.908336 (PMC9204228; doi:10.3389/fmicb.2022.908336)
Supplement: Supplementary file 1 [file Data_Sheet_1.zip › Data Sheet 1.PDF]

Witness questionnaire for scientists active in research on lactic acid bacterial bacteriocins from 1985 to 2005.

The objective of this questionnaire is to document the memories of individual researchers working with lactic acid bacterial bacteriocins in university laboratories during the 1990s and/or the 2000s. It is our intention that the questionnaire will constitute the core part of a publication in a special issue "Insights in Antimicrobials" in Frontiers in Microbiology.

We have divided the questionnaire into four parts concerning your profile, details regarding your work on bacteriocins, your general opinion on this field of research and your opinion on whether the research objectives were met.

Some questions concerns memories in your own work on bacteriocins. You most likely worked in a laboratory that included several researchers working on bacteriocins. If you worked with a narrow set of bacteriocins within the laboratory, e.g. as a master or Ph.D. student or as a Post-doc, you should include memories on these bacteriocins that were part of your own project. If you supervised post-docs and/or master or Ph.D. students, you may include all projects that fell within your supervision. If you worked with bacteriocins for an extended part of your career, you might have participated both as a junior, and a senior researcher within the same decade, such as the 1990s or the 2000s. In that case, you should combine your experience to give a general, balanced account.

Some questions concerns your opinion on the bacteriocin field as such. Regarding these questions you may draw on your overall memories ranging from those connected with your own work and those connected with the work by others, communicated to you by various formal and informal channels.

You may have worked with several different bacteriocin systems during your career; in that case you may respond in general terms in a way that represent your work in the most balanced way. You are encouraged to use the comment field to emphasize e.g. exceptions from your general account – e.g. to mention a bacteriocin that showed more promise for application in biopreservation than the other bacteriocins you worked with.

In overall, we encourage you to use the comment field to expand your answers as much as you wish. This may also constitute any additional information or insight based e.g. on personal experience or referring to published or unpublished materials, which help to obtain a more complete historical picture. All your answers are anonymous and complying with GDPR regulation. You are, however, most welcome to contact us directly for any inquiries, suggestions, or comments you might have. For this purpose, you may find our e-mail addresses listed in the cover letter.

We deeply appreciate your contribution and thank you for your generous use if time in completing the questionnaire.

The questionnaire consists of 48 questions and is expected to take approximately 20-30 minutes to complete, not considering the comments you may contribute.

A: Your profile

1. Your current age:
   30-40 41-50 51- 60 61-70 >70

2. Your years of research within the field of lactic acid bacterial bacteriocins:
   1-5 5-10 11-15 16-20 21-25 >25

3. Did you participate in research for new bacteriocins in the 1990s?

Yes: No: (if selected "no", questions related to the 2990s would not appear in the remaining part of the questionnaire).

4. What was your job position(s) at the time? You may here also indicate e.g. if you were a PI or you had a role as a collaborator (e.g. supplying expertise on taxonomy, chemical analyses etc.):

5. In which country(ies) did you perform your work at the time?

6. Did you participate in research for new bacteriocins in the 2000s?

Yes: No: (if selected "no", questions related to the 2000s would not appear in the remaining part of the questionnaire).

7. What was your job position(s) at the time? You may here also indicate e.g. if you were a PI or you had a role as a collaborator (e.g. supplying expertise on taxonomy, chemical analyses etc.):

8. In which country(ies) did you perform your work at the time?

B: Details on your research on bacteriocins

Which target organism(s) did you include in your bacteriocin research?

9a. Other lactic acid bacteria
Please, include genus (genera)/species or write “none” (if applicable) – you may insert a note if your selection of targets changed during your research:

9b. Gram positive pathogens in foods
Please, include genus (genera)/species or write “none” (if applicable) – you may insert a note if your selection of targets changed during your research:

10. Gram negative pathogens in foods

Please, include genus (genera)/species or write “none” (if applicable) – you may insert a note if your selection of targets changed during your research:

11. Food-borne spoilage microorganisms

Please, include genus (genera)/species or write “none” (if applicable) – you may insert a note if your selection of targets changed during your research:

12. Gram positive human clinical pathogens
Please, include genus (genera)/species or write “none” (if applicable) – you may insert a note if your selection of targets changed during your research:

13. Gram negative human clinical pathogens
Please, include genus (genera)/species or write “none” (if applicable) – you may insert a note if your selection of targets changed during your research:

14. Gram positive veterinary pathogens
    Please, include genus (genera)/species or write "none" (if applicable) – you may insert a note if your selection of targets changed during your research:

15. Gram negative veterinary pathogens
    Please, include genus (genera)/species or write "none" (if applicable) – you may insert a note if your selection of targets changed during your research:

16. Which bacterocin producing lactic acid bacterial genus/genera did you use in your study

Please, include genus (genera)/species or write "none" (if applicable) – you may insert a note if your selection of targets changed during your research:

17. Did you work with bacteriocin producers from other bacterial taxons than those included in the lactic acid bacteria?

    Please, include genus (genera)/species or write "none" (if applicable) – you may insert a note if your selection of targets changed during your research:

18. For which type of food, was the bacteriocin(s) intended for application as biopreservatives?

    Please, include type of food (e.g. MAP beef) or write "none" (if not applicable) – you may insert a note if type of food changed during your research:

19. Which type of bacteriocins did you include in your study? (Please, select all answers that apply)

Class I (lanthibiotics)
As metabolite, not purified: As partially purified compound: As purified compound:

Class IIa (small-heat stable peptides)
As metabolite, not purified: As partially purified compound: As purified compound:

Class III (proteins)
As metabolite, not purified: As partially purified compound: As purified compound:

Optional comment/elaboration (e.g. subclasses of class II or specific name(s) of bacteriocin(s))

20. If you conducted research on bacteriocins produced by other taxons than those included in the lactic acid bacteria, which type/class of bacteriocin(s) was the subject of study? You may also list the name(s) of bacteriocin(s).

I did not conduct research on bacteriocins produced by other taxons than those included in the lactic acid bacteria:

21. Was your research on bacteriocin(s) part of projects to find new probiotic cultures?
    Yes:
    No:

Comments:

22. What were your sources of lactic acid bacterial bacteriocin producers? (Please, select all answers that apply)

From external public culture collection(s):

From internal culture collection(s):

From culture collection(s) provided by company (e.g. a collaborator):

From other research groups in public research institutions (including universities):

Isolated as part of your research from the natural environment (e.g. vegetation, soil, water):

Isolated as part of your research from the built environment (e.g. a food processing plant):

Isolated as part of your research from various food products:

Isolated as part of your research from a specific category of food for which you aimed at applying a biopreservative culture:

Other:

I did not work with bacteriocin producers belonging to the lactic acid bacteria:

Comments:

23. What were your sources of bacteriocin producers other than lactic acid bacteria? (Please, select all answers that apply)

From external public culture collection(s):

From internal culture collection(s):

From culture collection(s) provided by company (e.g. a collaborator):

From other research groups in public research institutions (including universities):

Isolated as part of your research from the natural environment (e.g. vegetation, soil, water):

Isolated as part of your research from the built environment (e.g. a food processing plant):

Isolated as part of your research from various food products:

Isolated as part of your research from a specific category of food for which you aimed at applying a biopreservative culture:

Other:

I did not work with bacteriocin producers other than those belonging to the lactic acid bacteria:

Comments:

24. What were your sources of target organisms for bacteriocin producers? (Please, select all answers that apply)

From external public culture collection(s):

From internal culture collection(s):

From culture collection(s) provided by company (e.g. a collaborator):

From other research groups in public research institutions (including universities):

Isolated as part of your research from the natural environment (e.g. vegetation, soil, water):

Isolated as part of your research from the built environment (e.g. a food processing plant):

Isolated as part of your research from various food products:

Isolated as part of your research from a specific category of food for which you aimed at applying a biopreservative culture:

Other:

Comments:

25. To what extent was you initial search for new bacteriocins based on phenotypic empirical screening (e.g. deferred inhibition test or well assay)?

Scale:
1) To no extent
2) To a little extent
3) To some extent
4) To a high extent
5) To a very high extent

While you did active research in the 1990s

1. 2. 3. 4. 5. I do not remember:

While you did active research in the 2000s

1. 2. 3. 4. 5. I do not remember:

Comments:

26. To what extent did you include mode of action studies in your bacteriocin research?

Scale:
1) To no extent
2) To a little extent
3) To some extent
4) To a high extent
5) To a very high extent

While you did active research in the 1990s

1. 2. 3. 4. 5. I do not remember:

While you did active research in the 2000s

1. 2. 3. 4. 5. I do not remember:

Comments:

27. To what extent were the objectives listed below part of your mode of action studies (Please, select an answer for each objective).

Scale:
1) To no extent
2) To a little extent
3) To some extent
4) To a high extent
5) To a very high extent

a. Demonstrate bacteriocin applicability.
While you did active research in the 1990s

1. 2. 3. 4. 5. I do not remember:

While you did active research in the 2000s

1. 2. 3. 4. 5. I do not remember:

b. Patenting.
While you did active research in the 1990s

1. 2. 3. 4. 5. I do not remember:

While you did active research in the 2000s

1. 2. 3. 4. 5. I do not remember:

c. To obtain basic knowledge:
While you did active research in the 1990s

1. 2. 3. 4. 5. I do not remember:

While you did active research in the 2000s

1. 2. 3. 4. 5. I do not remember:

Other - please specify:

28. To what extent did you include genetic characterization of the genes involved in bacteriocin expression (such as genes encoding structural, regulatory, modification, transport aspects) in your bacteriocin research?

Scale:
1) To no extent
2) To a little extent
3) To some extent
4) To a high extent
5) To a very high extent

While you did active research in the 1990s

1. 2. 3. 4. 5. I do not remember:

While you did active research in the 2000s

1. 2. 3. 4. 5. I do not remember:

Comments:

29. To what extent were the objectives listed below part of your genetic characterization studies? (Please, select an answer for each objective)

Scale:
1) To no extent
2) To a little extent
3) To some extent
4) To a high extent
5) To a very high extent

a. Demonstrate bacteriocin applicability.

While you did active research in the 1990s

O——O——O——O——O
1. 2. 3. 4. 5. I do not remember:

While you did active research in the 2000s

O——O——O——O——O
1. 2. 3. 4. 5. I do not remember:

b. Patenting.

While you did active research in the 1990s

O——O——O——O——O
1. 2. 3. 4. 5. I do not remember:

While you did active research in the 2000s

O——O——O——O——O
1. 2. 3. 4. 5. I do not remember:

c. To obtain basic knowledge:

While you did active research in the 1990s

O——O——O——O——O
1. 2. 3. 4. 5. I do not remember:

While you did active research in the 2000s

O——O——O——O——O
1. 2. 3. 4. 5. I do not remember:

Other - please specify:

C: Details regarding your opinion on the field of bacteriocin research

30. In your opinion, how many research groups internationally had research on lactic acid bacterial bacteriocins as one of the primary topics in the 1990s?

Approximate number:

I do not know:

Comments:

31. In your opinion, how many research groups internationally had research on lactic acid bacterial bacteriocins as one of the primary topics in the 2000s?

Approximate number:

I do not know:

Comments:

32. According to your memory, how was your opinion at the time you did your research regarding:

The chances were high for finding new bacteriocins

Scale
1) Completely disagree
2) Strongly disagree
3) Somewhat disagree
4) Neither agree or disagree
5) Agree
6) Strongly agree
7) Completely agree

While you did active research in the1990s

○—○—○—○—○—○—○
1. 2. 3. 4. 5. 6. 7. I do not remember:

While you did active research in the 2000s

○—○—○—○—○—○—○
1. 2. 3. 4. 5. 6. 7. I do not remember:

Comments:

33. According to your memory, how was your opinion at the time you did your research regarding:

    The chances were high for finding new bacteriocins with potential practical applications for biopreservation of foods

Scale
1) Completely disagree
2) Strongly disagree
3) Somewhat disagree
4) Neither agree or disagree
5) Agree
6) Strongly agree
7) Completely agree

While you did active research in the 1990s

○—○—○—○—○—○—○
1. 2. 3. 4. 5. 6. 7. I do not remember:

While you did active research in the 2000s

○—○—○—○—○—○—○
1. 2. 3. 4. 5. 6. 7. I do not remember:

Comments:

34. According to your memory, how was your opinion at the time you did your research regarding:

The chances were high for finding new bacteriocins with potential practical applications for treatment of infections by human clinical pathogens

Scale
1) Completely disagree
2) Strongly disagree
3) Somewhat disagree
4) Neither agree or disagree
5) Agree
6) Strongly agree
7) Completely agree

1. 2. 3. 4. 5. 6. 7. I do not remember:

While you did active research in the 2000s

1. 2. 3. 4. 5. 6. 7. I do not remember:

Comments:

35. According to your memory, how was your opinion at the time you did your research regarding:

The chances were high for finding new bacteriocins with potential practical applications for treatment of infections by veterinary pathogens

Scale
1) Completely disagree
2) Strongly disagree
3) Somewhat disagree
4) Neither agree or disagree
5) Agree
6) Strongly agree
7) Completely agree

While you did active research in the 1990s

1. 2. 3. 4. 5. 6. 7. I do not remember:

While you did active research in the 2000s

1. 2. 3. 4. 5. 6. 7. I do not remember:

Comments:

36. According to your memory, how was your opinion at the time you did your research regarding:

    The chances were high for finding new practical applicable antimicrobial peptides from other organisms than lactic acid bacteria, including eukaryotic organisms (animals, plants and/or fungi) for medical treatment

Scale
1) Completely disagree
2) Strongly disagree
3) Somewhat disagree
4) Neither agree or disagree
5) Agree
6) Strongly agree
7) Completely agree

While you did active research in the 1990s

○—○—○—○—○—○—○
1. 2. 3. 4. 5. 6. 7. I do not remember:

While you did active research in the 2000s

○—○—○—○—○—○—○
1. 2. 3. 4. 5. 6. 7. I do not remember:

Comments:

37. According to your memory, how was your opinion at the time you did your research regarding:

    Target resistance to lactic acid bacterial bacteriocins was a cause for concern among researchers in the field.

Scale
1) Completely disagree
2) Strongly disagree
3) Somewhat disagree
4) Neither agree or disagree
5) Agree
6) Strongly agree
7) Completely agree

While you did active research in the 1990s

○—○—○—○—○—○—○
1. 2. 3. 4. 5. 6. 7. I do not remember:

While you did active research in the 2000s

○—○—○—○—○—○—○
1. 2. 3. 4. 5. 6. 7. I do not remember:

Comments:

38. According to your memory, how was your opinion at the time you did your research regarding:

    Lactic acid bacterial researchers were inspired by screenings for new antibiotics from especially fungi, *Bacillus* and *Streptomyces*

Scale
1) Completely disagree
2) Strongly disagree
3) Somewhat disagree
4) Neither agree or disagree
5) Agree
6) Strongly agree
7) Completely agree

While you did active research in the 1990s

1. 2. 3. 4. 5. 6. 7. I do not remember:

While you did active research in the 2000s

1. 2. 3. 4. 5. 6. 7 I do not remember:

Comments:

39. According to your memory, how was your opinion at the time you did your research regarding:

    Lactic acid bacterial researchers were inspired by studies on colicins

Scale
1) Completely disagree
2) Strongly disagree
3) Somewhat disagree
4) Neither agree or disagree
5) Agree
6) Strongly agree
7) Completely agree

While you did active research in the 1990s

1. 2. 3. 4. 5. 6. 7. I do not remember:

While you did active research in the 2000s

1. 2. 3. 4. 5. 6. 7. I do not remember:

Comments:

40. According to your memory, how was your opinion at the time you did your research regarding:

    Lactic acid bacterial researchers were inspired by contemporary screening studies in the related field of new animal, plant and/or fungal antimicrobial peptides

Scale
1) Completely disagree
2) Strongly disagree
3) Somewhat disagree
4) Neither agree or disagree
5) Agree
6) Strongly agree
7) Completely agree

While you did active research in the 1990s

1. 2. 3. 4. 5. 6. 7. I do not remember:

While you did active research in the 2000s

1. 2. 3. 4. 5. 6. 7. I do not remember:

Comments:

D: Details on your opinion on bacteriocin research objectives

41. In your opinion did your bacteriocin research in overall meet your objectives?

Scale
1) Completely disagree
2) Strongly disagree
3) Somewhat disagree
4) Neither agree or diagree
5) Agree
6) Strongly agree
7) Completely agree

While you did active research in the 1990s

1. 2. 3. 4. 5. 6. 7. I do not know:

While you did active research in the 2000s

1. 2. 3. 4. 5. 6. 7. I do not know:

Comments:

42. In your opinion did the overall field of research on lactic acid bacterial bacteriocins meet the objectives in terms of contributing to basic knowledge on bacteria antagonism?

Scale
1) Completely disagree
2) Strongly disagree
3) Somewhat disagree
4) Neither agree or diagree
5) Agree
6) Strongly agree
7) Completely agree

While you did active research in the 1990s

1. 2. 3. 4. 5. 6. 7. I do not know:

While you did active research in the 2000s

1. 2. 3. 4. 5. 6. 7. I do not know:

Comments:

43. In your opinion did the overall field of research on lactic acid bacterial bacteriocins meet the objectives in terms of contributing to practical applications in relation to biopreservation?

Scale
1) Completely disagree
2) Strongly disagree
3) Somewhat disagree
4) Neither agree or diagree
5) Agree
6) Strongly agree
7) Completely agree

While you did active research in the 1990s

1. 2. 3. 4. 5. 6. 7. I do not know:

While you did active research in the 2000s

1. 2. 3. 4. 5. 6. 7. I do not know:

Comments:

44. In your opinion did the overall field of research on lactic acid bacterial bacteriocins meet the objectives in terms of contributing to practical applications in relation to probiotics?

Scale
1) Completely disagree
2) Strongly disagree
3) Somewhat disagree
4) Neither agree or diagree
5) Agree
6) Strongly agree
7) Completely agree

While you did active research in the 1990s

○—○—○—○—○—○—○
1. 2. 3. 4. 5. 6. 7. I do not know:

While you did active research in the 2000s

○—○—○—○—○—○—○
1. 2. 3. 4. 5. 6. 7. I do not know:

Comments:

45. In your opinion did the overall research field on lactic acid bacterial bacteriocins meet the objectives in terms of contributing to practical applications in relation to human clinical microbiology?

Scale
1) Completely disagree
2) Strongly disagree
3) Somewhat disagree
4) Neither agree or diagree
5) Agree
6) Strongly agree
7) Completely agree

While you did active research in the 1990s

○—○—○—○—○—○—○
1. 2. 3. 4. 5. 6. 7. I do not know:

While you did active research in the 2000s

○—○—○—○—○—○—○
1. 2. 3. 4. 5. 6. 7. I do not know:

Comments:

46. In your opinion did the overall field of research on lactic acid bacterial bacteriocins meet the objectives in terms of contributing to practical applications in relation to veterinary microbiology?

Scale
1) Completely disagree
2) Strongly disagree
3) Somewhat disagree
4) Neither agree or diagree
5) Agree
6) Strongly agree
7) Completely agree

While you did active research in the 1990s

○—○—○—○—○—○—○
1. 2. 3. 4. 5. 6. 7. I do not know:

While you did active research in the 2000s

○—○—○—○—○—○—○
1. 2. 3. 4. 5. 6. 7. I do not know:

Comments:

47. If you compare your research in the 1990s and/or the 2000s with the bacteriocin research field today, please briefly describe your evaluation of contemporary importance of sequence-based methodology for screening for new bacteriocins and their subsequent structural and functional characterization:

Please, feel free to add any additional comment/memory/insight you feel is relevant to this questionnaire:

Thank you very much for taking your time to complete this questionnaire.
