# Supplementary material for: Scientists’ Assessments of Research on Lactic Acid Bacterial Bacteriocins 1990–2010
Source: Front Microbiol. 2022 Jun 3;13:908336. doi: 10.3389/fmicb.2022.908336 (PMC9204228; doi:10.3389/fmicb.2022.908336)
Supplement: Supplementary file 1 [file Data_Sheet_1.zip › Data Sheet 2.pdf]

Datasheet 2. List of respondents comments (with question number), including also two comments provided by e-mail. Authors comments in *italics*.

**Subclasses or specific name(s) of LAB bacteriocin(s) (Q19)?**
1) Often the compounds were not of sufficient value to classify them (especially back in the 1990s (Q19)
2) Nisin, macedocin, amylovorin, lactobin, sakacin, curvacin, enterocins (Q19)
3) Lantibiotics & class IIa - as metabolite, partially purified and purified (Q19)
4) Circular bacteriocins (Q19)
5) Sakacin A, sakacin P, nisin (Q19)
6) …better say RiPPs and use that nomenclature. Lasso peptides and sactipeptides and circular peptides etc. are also interesting for food applications Class II would be unmodified peptides (Q19)
7) Commercial nisin as control (Q19)
8) Encapsulation of nisin to make it work in minced meat (Q19)
9) Class IIb, nisaplin (Q19)
10) Divercin V41 (Q19)
11) Head-to-tail cyclized peptides (AS-48), nisin (Q19)
12) Carnobacteriocin (Q19)
13) Purified carnocyclin A from *C. maltaromaticum* UAL 307 for use in applied studies. Used as a metabolite, partially purified and purified compound.(Q19)
14) Worked mostly with pediocin-like sakacins (e.g. sakacin A and sakacin P). Worked with both non-purified, purified and synthetic preparations (not possible to give multiple answer for the same class). Often used nisin as control (hence the answer on lantibiotic above) (Q19)
15) For class I and II - all of the above (multiple selections not possible) (Q19)
16) My lab was only focused on understanding the mode of action of some lantibiotics, mostly nisin, on the cellular level. We described that these peptides have molecular targets, mostly lipid II and the complexity of cellular mechanisms triggered by binding to these targets. I never developed or tried to develop a lantibiotic for medical purposes since it is clear that cationic amphiphilic compounds in general have toxicity issues! (Q19)
17) Carnocin CP5, mesenterocin 52, curvaticin 13 (Q19)
**Subclasses or specific name(s) of non-LAB bacteriocin(s) (Q20)?**
18) Class 1, class2, class 3 (Q20)

19) Class II (Brochocin-C) (Q20)
20) Thuricin H, Matticin A (Q20)
21) Micrococcin (*Mammaliicoccus*) (Q20)
22) Class I, class IIa (Q20)
23) Enterococcins, listeriocin, bifidobacteriocins (Q20)
24) Bacilli producing RiPPs, NRPS and PKS (Q20)
25) Lantibiotics, thiopeptides (Q20)
26) All different types but some examples are thuricin CD from *Bacillus thuringinsis*, nisin J from *Staph*. *capitas* but there is an extensive list (Q20)
27) From *Bacillus* (Q20)
28) Colicin V, Microcin N (Q20)
29) *Bacillus pumilus* (pumilicin) (Q20)
30) Class I (Q20)
31) Class II bacteriocins, warnericin RK, delta-hemolysins and phenol soluble modulins (Q20)
32) Lantibiotic & protein (Q20)
33) Lantibiotics (Q20)
34) Class I, lantibiotic; nukacin ISK-1 (Q20)
35) From *Bacillus* (Q20)
**Isolation of bacteriocinogenic cultures? Screening and identification of bacteriocins including rediscoveries? New classes of AMP? (see also comments 148, 150 and 155) – one comment as an e-mail**
36) Often the compounds were not of sufficient value to classify them (especially back in the 1990s (Q19)
37) Many individual screening projects on many different sources (Q22)
In the 1990's the bacterium was part of my supervisor's collection. As a PI, I used commercial preparations (Q22 and Q23)
38) Isolated as part of our research from the natural environment (cows' teat apex)(Q23)
39) Bacilli from soil and leaves (Q23)
40) The (*bacteriocin name*) work was due to the very broad spectrum of activity of this bacteriocin (Q25).
41) Used mostly already described bac-producers (Q25)
42) PCR was also used but for new bacteriocins mostly emperical screening (Q25)
43) No screening at all (Q25)

44) All my work to search for new bacteriocins has been carried out via the well diffusion method (Q25)
45) I found something novel (… *a hydrophobic, negatively charged molecule*) but it was not a bacteriocin. (Q32)
46) Agree (*Chances were high for finding new bacteriocins)* but some people kept finding the original pediocin again and again! (32)
47) We found several new bacteriocins (Q32)
48) But I was wrong (*regarding chances were high for finding new bacteriocins*) (Q32)
49) Screening procedure (in laboratory media first) was not adequate (Q41)
50) Hundreds of new bacteriocins have been reported. But good characterization lacks in most papers…finding a (partly) new variant or new bacteriocin and test it directly in application without thorough thinking of required physico-chemical properties, toxicity, target specificity, bioavailability, etc. (Q48)
51) I go almost as far back as 1990... I … still remember NN urging to put less emphasis on looking for new bacteriocins as most of the diversity was already characterized - that may not have been quite correct in 1996 but, a few hundred genomes later, certainly was in 2010 – 2015 (comment provided by e-mail by one respondent)
52) AMPs also include NRPS PKS?? Or only RiPPs? What is meant here? Is a natural variant of e.g. nisin a new peptide?? Are engineered peptides (being screened) also considered new peptides. For food is problematic (GMO problem) but for pharma use is relevant (Q36)
**Mode of actions (Q26, Q27)?**
53) We didn't do any proper mode of action studies (Q26)
54) See comments before on the aims of my research -- understanding fully the mode of action of antibiotics is necessary for the design of antibiotics particularly because it became clear as a result of our research that host defense peptides and even classical antibiotics such as glycopeptides follow the same route of activities (Q23) *Previous comment: "My lab was only focussed on understanding the mode of action of some lantibiotics, mostly nisin…" (see comment 16)*
55) Development of resistance (Q27) In response to *"To what extent were the objectives listed below part of your mode of action studies"*
**Genetic characterizations (Q28, Q29)?**
56) In one study the aim was to compare gene clusters responsible for sakacin production in different strains (Q28)
57) Regulation of bacteriocin production (Q29)
**GMOs?**
58) We made lantibiotics in *E. coli* from synthetic genes (Q23)
**Virulence/antibiotic resistance of bacteriocin producers?**
59) Actually recently, a large majority of strains revealed to be antibiotic resistant and research aborted since the aim was to select bioprotective cultures (Q22)

60) …more recently, the major problem was the carriage of antibiotic resistance genes by the putative producers that led to abandon the screening, whatever it was performed in laboratory or under food conditions (Q41)
**Target resistance (Q26, Q37)?**
61) I created bacteriocin-resistant mutants (*Listeria monocytogenes*) that were no longer susceptible to specific bacteriocins (if bacteriocin resistance to one bacteriocin knocked out sensitivity to another bacteriocin, I considered them to have the same mode of action); I then used that bacteriocin-resistant *L. monocytogenes* strain as an indicator for additional bacteriocins that would indicate a different mode of action. After several sequential series of these, I obtained about 3-4 different groupings of bacteriocin that I considered to have different modes of action and used them in aggregate to achieve high efficiency inhibition (Q26)
62) It was an important part of our research and application for use in sliced deli Meat. Lots of discussion with authorities (Q37)
63) Only as much as bacteriocins would be used as biopreservatives, then resistance to bacteriocins would be a concern that would diminish their effectiveness (Q37)
64) It was not a cause of concern - BUT it should have been a major concern (Q37)
**Number of research groups (1990s) (Q30)?**
65) Many or most groups working on LAB at the time also worked on bacteriocins (Q30)
66) This is mostly a guess but I'd say maybe 40 labs were serious bacteriocin researchers (Q30)
67) Almost impossible to answer this question! So many did empirical screening studies; far fewer did detailed molecular/genetic/MoA characterization (Q30) – *same reply for 2000s* (Q31)
68) Several also in Korea, Japan, China (Q30)
69) Many! (Q30)
70) There were some 10-20 top teams and when this research became popular hundreds of teams jumped on the train (Q30)
**Number of research groups (2000s) (Q31)?**
71) But less than in the 90s I think (Q31)
72) Interest in bacteriocins dropped in the early 2000 (Q31)
73) Less than in the 1990s (Q31)
**Research inspired by earlier research on antibiotics (Q38)?**
74) I don't think this was the focus very much (Q38)
75) I presumed there would be a concern that you might be finding antibiotics rather than bacteriocins from those organisms (Q38)
**Research inspired by earlier research on colicins (Q39)?**
76) We did some of this but overall this was a separate field in my opinion (Q39)

**Objectives reached for own research (Q41)?**
77) For me the science and the training was very good and that was my real objective at the time (Q41)
78) No; difficult to find anything 'better' than nisin; most promising candidates had poor yield making commercialization very challenging (Q41)
79) In both research projects some of the bacteriocin producing lactic acid bacteria isolated were patented and commercialized (Q41)
80) My objective was mainly fundamental; research and understanding biosynthesis and mode of action. Later in 2000-2022 it was more directed to engineering RiPPs to fight Gram negative human pathogens (Q41)
81) My objectives were learning about how potent antibiotics kill on the molecular and cellular level - and for this we learned a lot from nisin and other lipid II targeting compounds (Q41)
**Objectives reached for whole field: Basic science (Q42, e-mail)?**
82) There was a lot of very good research done in terms of basic science. (Q42)
83) What is still lacking after more than 30 years of bacteriocin research is a convincing cause of why bacteriocins matter for bacterial ecology (Q48)
84) …maybe the limited activity range of many bacteriocins was an issue (often towards related strains/ species), the difficult isolation of the active molecules (hydrophobic), the resistance mechanisms and maybe also the link with the GMO nature (most likely) of the final applications (comment provided by e-mail by one respondent)
**General comment on application difficulties**
85) One of the main drawbacks of bacteriocin research is the difficulties that are met when the potential (more easily demonstrated by many different labs around the world) need to translate into real applications due to the research cost and limitations imposed by public funding. Private investors show no real interest until extensive (and expensive) toxicity studies are available (Q48)
**Application of bacteriocins as food biopreservatives (Q33)? Objectives reached for whole field: Food biopreservation (Q43) including difficulties for funding?**
Memories of opinions in 1990s and/or 2000s
86) I was never very confident they would work practically (Q33)
87) I don't think bacteriocins have had much success for practical applications although I may be wrong (Q43)
Current opinions
88) There was some development on the use of protective cultures in the food industry but not as extensive as first expected (Q43)
89) I don't think bacteriocins have had much success for practical applications although I may be wrong (Q43)
90) But the problems with resistance to bacteriocins and the problems with accepting high bacterial count in products traditionally produced with low bacterial numbers has been a challenge hard to overcome; To succeed in finding bacteriocins suitable for

biopreservation attention must be on legislation. How can the challenge with resistance and high bacterial numbers in for example sliced deli meat be solved (Q43 and Q47)
91) Not many great successes except for nisin and a few other peptides (Q43)
92) There were many reports about the potential of LAB bacteriocins but the number of compounds approved for this use did not grow accordingly (Q43)
93) Not so far - there is still some potential but development seems not economically promising (43)
94) Again not yet - for economic reasons (*response to "did the overall field of research on lactic acid bacterial bacteriocins meet the objectives in terms of contributing to practical applications in relation to probiotics?"*)(Q47)
95) Happy to see the success of *Carnobacterium maltaromaticum* as a bioprotective culture (Q48)
96) Food industry is reluctant to make changes to incorporate bacteriocin technology to improve food safety (Q48)
97) The matrix effects and low activity in foods, as well non-acceptance by the consumer (discussion that bacteriocins are antibiotics) severely dampened the application of bacteriocis as biopreservatives (Q48)
**Application of bacteriocins as enhancers of probiotic actions (various questions)? Objectives reached for whole field: Probiotics (Q21, Q44)?**
98) Some projects were, but many were simply looking for antimicrobials (Q21)
99) Sometimes (Q21) *In response to "Was your research on bacteriocin(s) part of projects to find new probiotic cultures?"*
100) Not at the time, but it is now (Q21)
101) Only a minor part. Most was engineering a known one like nisin and screen mutant libraries (not for food though) (Q21)
102) Research was focused on biopreservation, not really on bacteriocins (Q21) *In response to "Was your research on bacteriocin(s) part of projects to find new probiotic cultures?"*
103) We should find applications for the known ones. The regulatory homologation is a major hurdle ((Q21)
104) But not exclusively (Q21) *In response to "Was your research on bacteriocin(s) part of projects to find new probiotic cultures?"*
105) Partially, not all bacteriocin research was in the context of probiotics (Q21)
106) I don't think this has happened. (Q44)
107) The documentation on probiotic effect is so high that I do not think many strain passed for this application (Q44)
108) There are a few (Q44)
109) Bacteriocin activity was impossible to prove to be probiotic relevant as was it difficult to prove that lactic acid bacteria probiotics had probiotic activity (human studies, meta studies) (Q44)
110) …Not yet - for economic reasons (Q44)

**Application of bacteriocins (and AMP in general) in human clinical microbiology (Q34)? Objectives reached for whole field: Human clinical microbiology (Q34, Q45)?**
Memories of opinions in 1990s and/or 2000s
111) Same as for food. I was never very confident in this approach. (Q34)
112) …I never developed or tried to develop a lantibiotic for medical purposes since it is clear that cationic amphiphilic compounds in general have toxicity issues! (Q19 and Q36)
113) We never looked into this. We were looking for applications in food and they should not at the same time be used for clinical purposes (Q34)
114) *(In)* my memory the interest was more focused on food protection than on medical applications (Q34)
115) Bacteriocin applicability for human therapy at that time was almost non-existing. Only a few reports suggested the use of bacteriocins for this, and they were mostly focused on lantibiotics. Pharmacokinetic studies were really scarce, only about duramycins (*produced by Streptomyces griseoverticillatus*) as far as I recall and aimed at cystic fibrosis treatment and not antimicrobial therapy (Q34)
Current opinions
116) I don't think this has happened (Q 45)
117) Clinical use requires a lot more: low toxicity, stability in blood, PK/PD, bioavailability etc. LAB bacteriocins as such are not very suitable in my opinion. Engineered variants with altered properties could have potential (Q45)
118) Non known application in clinical microbiology as far as I know (Q45)
119) There are toxicity issues with systemic treatment, simply not doable - for topical treatment there is not enough medical need - if resistance problems continues to rise and research on human microbiome provides better basis for rational application of probiotic strains this may become an option - however current ongoing developments of new beta- lactams/combinations of beta-lactams and beta-lactamase inhibitors via classical medicinal chemistry will bring new options for antibiotic treatment in the near and make development of bacteriocin-producing strains for medical treatments not economically feasible (Q45)
**Application of bacteriocins in veterinary microbiology (Two comments also on feed additives; Q20, Q27)? Objectives reached for whole field: Veterinary microbiology (various questions) (Q35, Q46)?**
120) It was a commercial probiotic feed additives, so known to the company only (Q20) *Answer provided to "Subclasses or specific name(s) of non-LAB bacteriocin(s)?"*
121) Application as feed additives. Application in meat products (Q27)
122) This wasn't my area but same as the others (Q35)(see comment 86 and 111)
123) According to my memory the interest was more focused on food protection than on veterinary applications (Q35)
124) Some reports on lantibiotics appeared, such as cattle mastitis treatment (Q35)

125) To small extent. Teat seals for instance (Q46)
**Impact by sequence-based methodology (Q47)?** *Both memories of opinions in 1990s and/or 2000s and current opinions*
126) Vital in both periods, used more as an *in silico* screening method in the 2000s (Q47)
127) Genome sequencing is a game changer - it makes it so much easier to screen for potential bacteriocin producers that also have other beneficial properties. It does not necessarily detection of something new easier as unknown genes / metabolic functions are not annotated correctly...(Q47)
128) I think that the sequence-based methodology has sped up the initial screenings (Q47)
129) Sequence data is fine but you still have to do experiments! (Q48)
130) I became less involved when these new methodologies (including bioinformatics) became available and easily applied but clearly they had a big impact (Q47)
131) Sequencing can provide a much faster answer to determining if the bacteriocin is novel compared to the lengthy process of purification, Edman degradation, or 3D NMR structural determination (Q47)
132) The tools have become much more powerful (Q47)
133) I was mostly involved in basic structure function studies of bacteriocins. I dont think the bacteriocin research area has made a big jump since then. Maybe the research today is more directed toward application and resistance compared to my period as bacteriocin researcher (Q47)
134) Understanding the genetic sequence aspects of bacteriocin production is important to understanding best use under a variety of conditions (Q47)
135) Very important to distinguish new isolates from existing bacteriocins (Q47)
136) Sequence based methodology is great importance and will hopefully drive this area of research forward more rapidly (Q47)
137) Genome based screening speeds up detecting the potential production of existing bacteriocin classes but still requires considerable functional analysis with technologies that are receiving limited attention.... new bacteriocins may be missed (Q47)
138) High-throughput screening methods and novel bioinformatic approaches to finding gene clusters in (meta) genome data open up a whole new range of possibilities. Also plug-and-play (synthetic biology) methods and high-throughput proteomic technologies should give a boost to bacteriocin research. Notwithstanding this, the research on application potential is lagging behind, because of a lack of tools keeping up with the speed of bacteriocin discovery. Also, funding for this kind of research seems to become a problem (Q47)
139) 1990's involved more phenotypic comparisons, enzymatic inactivation, protein purification & partial sequencing. 2000's I was able to utilize published sequences to identify PCR primers to use to screen organisms and identify bacteriocin sequences without having any information of DNA sequence of the unknown/new bacterial isolate (Q47)
140) I do not closely follow the bacteriocin field today, but whole genome sequencing makes it easier to find bacteriocin candidates (Q47)

141) There are lots of sequences, but difficult to figure out which ones are of interest (Q47)
142) More opportunities now a days. But screening might not be the primary solution. Finding or developing compounds with the right characteristics is more important (Q47)
143) I saw a recent paper that convinced me that genome mining and other omics as proteomics or metabolomics should bring new information for discovering new antimicrobial compounds, including bacteriocins (Q47)
144) Metagenomics analysis can point out easily potential bacteriocin producers (Q47)
145) Sequence-based methodology has increased rapid screening of new bacteriocins and improved insight into the maturation mechanisms of peptides (Q47)
146) Large scale plate screening metagenomic sequencing gene trait matching BAGEL and AntiSMASH (Q27)
147) Sequence-based methodology is becoming increasingly important to structural and functional characterization; for screening, it allows to orient the selection strategy (producer and target strain)(Q47)
148) With the phenotypic detection of bacteriocins, there was a great risk to miss bacteriocin producers because of specific production conditions or regulation of some bacteriocins. Discovering a new bacteriocin with interesting inhibition properties and significant activity was a luck. The sequence based methods offers both the opportunity to find new bacteriocins, but also to identify quickly already characterised bacteriocins (Q47)
149) The field grew in the last years with many reports on genome sequencing, mining (e.g. BAGEL, AntiSMASH), and biotechnology/synthetic biology approaches. Mass-spectrometry has also contributed to reduce dereplication and characterization of modification patterns (e.g. lantibiotics, sactipeptides and other RiPPs with antimicrobial activity) (Q47)
150) Sure, it is much easy to find new bacteriocins nowadays due to the advent of genome sequencing technologies. Also synthetic DNA and synthetic peptides (Q47)
151) Highly important but nothing beats an inhibition assay to ensure a peptide is active (Q47)
152) Sequence-based methodology could have a large potential. However, considering the very large effort that was done in the 1990s (and beginning of 2000s) without any major breakthrough with regard to applications in food preservation or medicine, I have my doubts. If there are very useful bacteriocins out there, we should perhaps have found them by now, even without sequence-based methodology. Perhaps targeted engineering of known bacteriocins/antimicrobial peptides is another way to go? Or perhaps both, in parallel? (Q47)
153) Apart from genome sequencing and mining, not much has changed. Challenges are resistance, low yields and protease digestion (Q47)
154) It is difficult to find new one (Q47)
155) It has reduced the time in the search for new bacteriocins (Q47)

156) …In my experience detecting bacteriocin genes has little relationship with the potential of a strain as a producer or biopreservative agent (Q47)
157) This approach became important in the mid-2000s but has become much important now (with cheaper sequencing and larger databases) (Q47)
158) Sequence based methods are very important for screening new bacteriocins (mining). Nevertheless the activity must be tested still in physiological tests (Q47)
159) I don't think that any kind of screening, including sequence will identify bacteriocins or antimicrobial peptides in general will identify better compounds than those we have already at hand and which we did not develop further for reasons I mentioned before (*See section above about funding*) (Q47)
160) Very important (Q47)
161) It enabled us to find out new bacteriocins in shorter time (Q47)
162) Sequence based methodologies are very important (Q47)
