# Supplementary material for: Scientists’ Assessments of Research on Lactic Acid Bacterial Bacteriocins 1990–2010
Source: Front Microbiol. 2022 Jun 3;13:908336. doi: 10.3389/fmicb.2022.908336 (PMC9204228; doi:10.3389/fmicb.2022.908336)
Supplement: Supplementary file 1 [file Data_Sheet_1.zip › Tables 1-7.pdf]

Suppl. tables

Suppl. Table 1. Age range and years of research for the respondents (Q1 and Q2).

Age range	Number of respondents	Years of research within the field of lactic acid bacterial bacteriocins	Number of respondents
30-40	0	1-4	2
41-50	5	5-10	11
51-60	24	11-15	7
61-70	18	16-20	7
>70	1	21-25	7
		>25	14

Suppl. Table 2. Distribution among countries of research done by respondents (Q5 and Q8).

County	1990s	2000s
Belgium	2	1
Brazil	0	1
Bulgaria	1	0
Canada	8	2
Denmark	2	4
Germany	2	2
Greece	1	1
France	7	4
Ireland	4	4
Italy	1	0
Japan	1	2
Netherlands	6	3
New Zealand	1	1
Norway	7	3
Malaysia	0	1
Portugal	1	0
South Africa	2	1
Spain	2	3
U.S.A.	5	4
Total	53	37

Suppl. Table 3. Number of respondents who used LAB bacteriocin producers in their research (Q16)

Genus or species	Number	Genus or species	Number
Carnobacterium sp.	6	*Lactococcus* sp.	9
Carnobacterium divergens	5	*Lactococcus garviae*	1
Carnobacterium inhibens	1	*Lactococcus lactis*	12
Carnobacterium maltaromaticum	10	*Lactococcus piscium*	1
Enterococcus sp.	6	*Leuconostoc* sp.	3
Enterococcus durans	1	*Leuconostoc carnosum*	1
Enterococcus faecalis	2	*Leuconostoc gelidum*	4
Enterococcus faecium	6	*Leuconostoc mesenteroides*	4
Enterococcus mundtii	1	*Pediococcus* sp.	5
Lactobacillus sp.	15	*Pediococcus acidilactici*	3
Fructilactobacillus	1	*Pediococcus cerevisiae*	1
Fructilactobacillus sanfransiscensis	1	*Pediococcus pentosaceus*	3
Lacticaseibacillus casei	1	*Streptococcus* sp.	2
Lactiplantibacillus plantarum	7	*Streptococcus pyogenes*	1
Lactobacillus acidophilus	3	*Streptococcus thermophilus*	1
Lactobacillus amylovorus	1	*Weissella paramesenteroides*	1
Lactobacillus delbrueckii	1	*Weissella viridescens*	1
Latilactobacillus curvatus	5		
Latilactobacillus sakei	8	"all of them"	1
Levilactobacillus brevis	1	"many others in screenings"	1
Limosilactobacillus	1	"none"	1

Suppl. Table 4. Number of respondents who used non-LAB bacteriocin producers in their research (Q17).

Genus or species	Number	Genus or species	Number
Bacillus sp.	4	*Paenibacillus* sp.	1
Bacillus halodurans	1	*Paenibacillus polymyxa*	2
Bacillus licheniformis	2	*Paenibacillus terrae*	1
Bacillus subtilis	4	*Salmonella enterica*	1
Bacillus thuringiensis	1	*Staphylococcus* sp.	4
Bifidobacterium sp.	1	*Staphylococcus agneti*	1
Brochothrix campestris	2	*Staphylococcus aureus*	1
Clavibacter sp.	1	*Staphylococcus epidermis*	1
Escherichia coli	1	*Staphylococcus lugdunensis*	1
Mammaliicoccus sp.	1	*Staphylococcus warneri*	2
Mammaliicoccus sciuri	1	"none"	26

Suppl. Table 5. Number of respondents who used non-pathogenic LAB target organisms in their research (Q9a).

Genus or species	Number	Genus or species	Number
Carnobacterium sp.[a]	5	*Leuconostoc sp.*[a]	8
Carnobacterium maltaromaticum	4	*Leuconostoc carnosum*	1
Carnobacterium divergens	3	*Leuconostoc mesenteroides sp.*[a]	2
Enterococcus sp.[b]	9	*Leuconostoc paramesenteroides*	1
Enterococcus faecalis[b]	5	*Limosilactobacillus sp.*	1
Enterococcus faecium[b]	6	*Pediococcus sp.*	4
Enterococcus mundtii	1	*Pediococcus acidilactici*	1
Fructilactobacillus sp.	1	*Pediococcus pentosaceus*	2
Fructilactobacillus sanfranciscensis	1	*Streptococcus sp.*	2
Lactiplantibacillus plantarum	6	*Streptococcus thermophilus*	2
Lactobacillus sp.[c]	11	*Tetragenococcus halophilus*	1
Lactobacillus acidophilus	2	*Weissella viridescens*	1
Lactobacillus delbrueckii	3		
Latilactobacillus curvatus	1	None	7
Latilactobacillus sakei[a]	6	LAB in general	8
Lactococcus sp.	6	Do not remember	1
Lactococcus lactis	9		

[a]1-2 researchers listed these taxons under spoilage organisms but without listing them as LAB target strains. They are added to this table.

[b]Six researchers listed enterococci under pathogenic Gram positive target strains but without listing them as LAB target strains; They are added to this table, except for vancomycin resistant enterococci target strains used by one researcher.

[c]Presumably including species now reallocated to other genera (Zheng et al 2020).

Suppl. Table 6. Number of respondents who used various non-LAB target organisms in their research.

**Gram positive foodborne pathogens (Q9B)**		**Gram negative foodborne pathogens (Q10)**[f]	
Bacillus sp.	3	*Campylobacter jejuni*	5
Bacillus cereus	9	*Escherichia coli*[g]	16
Clostridium sp.	2	*Salmonella enterica*	11
Clostridium botulinum	2	*Salmonella* Typhimurium	3
Clostridium perfringens	3	None	24
Clostridium sporogenes[a]	2	**Gram positive food spoilage organisms (non-LAB) (Q11)**	
Enterococcus sp. (VRE)[b]	1	*Alicyclobacillus* sp.	1
Listeria sp.[c]	14	*Arthrobacter* sp.	1
Listeria innocua[d]	3	*Bacillus* sp.	6
Listeria monocytogenes	28	*Brochothrix* sp.	1
Staphylococcus sp.	9	*Brochothrix thermosphacta*	5
Staphylococcus aureus	11	*Clostridium* sp.	3
Streptococcus sp.	4	*Clostridium algidicarnis*	2
Streptococcus agalactiae	1	*Clostridium estertheticum*	2
Streptococcus mutans	1	*Clostridium frigidicarnis*	2
Streptococcus pyogenes/group A[e]	2	*Clostridium tyrobutyricum*	4
		Paenibacillus sp.[h]	1
		**Other Gram positive organisms**	
		Brochothrix campestris	1
		Clavibacter sp.[i]	1
		Micrococcus flavus	1
		Micrococcus luteus	3

[a]One respondent informed on use of *Cl. sporogenes* as model target for *Cl. Botulinum*, another included it under Q11.

[b]Listed here due to the antibiotic resistant profile

[c]Including two respondents who listed this pathogen under spoilage organisms (Q11)

[d]Model target for *L. monocytogenes*

[e]For *S. pyogenes* as a foodborne pathogen, see Falkenhorst et al. 2008.

[f]Most likely in nearly all cases included as control target organisms although only specified by one respondent (acid inhibition)

[g]Most target strains probably non-pathogenic including one respondent who used *E. coli* as target for colicin V. Pathogenic variants informed by three respondents.

[h]One species is an insect pathogen

[i]Include plant pathogenic species

Suppl. Table 7. Types of foods included in respondents' research for applying bacteriocins for biopreservation (Q18)

Product	Number	Product	Number
Drinks, juice, produce, salad dressings	6	Milk and dairy products	19
Fermented foods (not meat and seafood)	3	- Cheese	8
Meat	35	Seafood	10
- Ready-to-eat	17	Sourdough	2
- Poultry, liquid egg	3	Not done/not listed	10
